# Job strain, social support, and alcohol-related health problems: A register-based cohort study

**DOI:** 10.5271/sjweh.4257

**Published:** 2026-01-01

**Authors:** Emelie Thern, Erica Jonsson, Devy L Elling, Melody Almroth

**Affiliations:** 1 Institute of Environmental Medicine, Karolinska Institute, Stockholm, Sweden.

**Keywords:** job demand, job control, Sweden, work environment

## Abstract

**Objective:**

This study aimed to examine the association between job strain (combination of job demands and job control) and alcohol-related health problems among men and women and the extent to which workplace social support moderates this association.

**Methods:**

This study used information from the register-based Swedish Work, Illness, and Labor-market Participation (SWIP) cohort, focusing on working individuals born 1945–1975, who were registered in Sweden in 2005 (N=2 822 462). Job demands, control and workplace social support were measured using job exposure matrices (JEM). Information on alcohol-related health problems (morbidity, mortality, medication prescription, sickness absence and disability pension) was obtained from multiple registers between 2006 and 2020. Cox regression models were employed to estimate associations between job strain and alcohol-related health problems, adjusting for sociodemographic background and previous health. The modifying effect of social support was assessed using relative excess risk due to interactions (RERI).

**Results:**

High-strain and passive jobs were related to an increased risk of alcohol-related health problems among both men [hazard ratios (HR) 1.28 and 1.32] and women (HR 1.06 and 1.05), after adjusting for important individual and parental covariates. Weak social support had diverging associations with the outcome for men and women. Strong social support appeared to buffer the risk in passive jobs but only among women (RERI=0.08).

**Conclusions:**

In Sweden, job strain seems to influence alcohol-related health problems, particularly among men. Women, however, appear to experience the protective effects of strong work-related social support measured at the occupational level.

Alcohol consumption can have detrimental consequences in multiple domains of everyday life. One such domain is the workplace through productivity loss due to sickness absence, sickness presenteeism, poor performance, and occupational injuries (1, 2). A significant share of Swedish workers (around 20%) report hazardous alcohol use (3), which is a pattern of drinking that can put individuals at risk for adverse health consequences (4). Occupational differences in the prevalence of hazardous alcohol consumption and alcohol-related health problems have previously been established (5–7), but the role of the workplace in explaining these differences remains unclear.

One perspective is that the workplace influences employees’ alcohol consumption through the alcohol norms and alcohol availability at work, such that less restrictive drinking norms and alcohol being more easily available increases the consumption of alcohol (6, 8, 9). Another perspective is that the psychosocial work environment could influence employees’ alcohol consumption through tension reduction, such that employees drink alcohol to deal with stress and negative emotions arising from poor psychosocial work environments (10).

A commonly used measure of the psychosocial work environment is the job demand–control–support model (11). The basis of this model is that high job demands combined with low control are associated with poor health outcomes, especially if workplace social support is also low (11). Job strain, a measure of work stress, is defined as the balance between job demands (ie, time pressure, workload) and job control (ie, decision latitude to control and plan work activities) (12, 13). High-strain jobs (high demand and low control) have previously been linked to heavy drinking (12) and alcohol-related morbidity (14). Workplace social support has been proposed to help employees cope with their job strain (11). Previous research has found that low levels of support are associated with an increased use of alcohol (15–17). Only a few studies have investigated if workplace social support influences the association between job strain and alcohol consumption (18, 19). The results of these two studies suggest that workplace social support reduces the impact of job strain on self-reported alcohol consumption. However, less is known about the influence of workplace social support on more objective alcohol-related consequences, such as morbidity, disability pension, and mortality.

Furthermore, only a few studies have investigated the difference among men and women with regards to the association between the psychosocial work environment and alcohol-related health problems (14, 17, 20). The effects of a poor psychosocial work environment also appear to be more pronounced among men compared to women in relation to alcohol dependence (20) and alcohol-related morbidity diagnosis (14). In contrast, a Canadian study found that higher psychological demands lead to less drinking for men and more drinking for women (17). While this study measured workplace social support and found that low social support was correlated with higher alcohol use across the entire population, it did not examine sex differences in relation to the effects of social support (17).

One previous study using the same cohort as the present study investigated the association between job-strain and alcohol-related morbidity identified in the Swedish national patient register (14). Using only the patient registers may underestimate alcohol-related problems among women as women are more likely to receive alcohol-related sickness absence rather than specialist or hospital diagnoses (21). Consequently, studies using only patient diagnoses may not fully reflect women’s alcohol-related health issues. Moreover, the potential moderating role of workplace social support in this association remains insufficiently studied. The current study aims to examine the association between job strain (combination of job demands and job control) and a variety of alcohol-related health problems among men and women, and the extent that workplace social support moderates this association.

## Method

### Study population

The study uses the register-based Swedish Work, Illness, and Labor-market Participation (SWIP) cohort which includes everyone registered in Sweden aged 16–64 years in 2005 (around 5.4 million people) (22).

In the current study, we included individuals who were born between 1945 and 1975 (age 30–60 in 2005) as these individuals were presumably established in the labor market (N=3 806 948). Individuals were excluded if they had missing information on occupation at baseline (N=787 489), registered alcohol-related morbidity before baseline (N=33 899) or were on disability pension at baseline (N=163 098). Compared to the included study participants, a higher proportion of the excluded individuals were born outside of Sweden, had lower educational attainment and more mental health problems (supplementary material, XXX, table S1). The final analytical sample consisted of 2 822 462 individuals.

### Data sources

Several linked data sources were used. The Longitudinal Integrated Database for Health Insurance and Labour Market Studies (LISA) provide annual data on sociodemographic characteristics, education, employment, and income (23). The National Patient Register (inpatient care and specialized outpatient care) and Cause of Death Register contain information on diagnoses according to the Swedish version of the International Classification of Diseases (ICD) versions 8, 9 or 10. The MiDAS register contains information on date and diagnoses of sickness absence and disability pension. The National Prescribed Drug Register contains information on all prescribed and dispensed medications in Sweden, including drug type, dosage, and date of dispensation. Lastly, the Multi-Generation Register (MGR) includes information on biological and adoptive parents.

### Variables

*Job control, job demands, and workplace social support.* Information on psychosocial exposures in the workplace, specifically job control, job demands, and workplace social support was obtained using the psychosocial job exposure matrix (JEM). This JEM is based on data from the Swedish Work Environment Surveys, which involves around 90 000 participants across approximately 350 occupations from 1997 to 2013. The JEM comprises of sex-specific index means for each psychosocial exposure within each occupation. Using this information, we were able to link the sex-specific index mean of each psychosocial exposure to the study participants’ occupation at baseline using the Swedish ISCO-88 four-digit classification of occupations. The questions used to measure job control, job demands, and workplace social support can be found in supplementary table S2. These JEM have been widely applied in previous research across various outcomes (22, 24), and an earlier version has been validated (25).

*Job control* was determined through seven questions related to decision-making authority (reflecting how much influence individuals have over their work processes) and skill discretion (concerning opportunities for learning and development of skills). *Job demands* were assessed using three questions that focused on aspects such as stress, time pressure, and the level of concentration required. *Job strain* is characterized by the combination of job control and job demands, divided into two groups using the sex-specific median as the cutoff point, which is in line with the job demand–control model and previous research (11, 14, 24). This analysis resulted in four classifications: passive jobs (low demands and low control), low-strain jobs (low demands and high control), active jobs (high demands and high control), and high-strain jobs (high demands and low control).

*Workplace social support* was determined through the average score of five questions related to support from supervisors and colleagues for men and women separately and then dichotomized into a sex-specific median to define strong and weak levels of support.

### Alcohol-related health problems

Information on the outcome of alcohol-related health problems between 2006 and 2020 was collected from multiple nationwide registers. The National Patient Register (inpatient care and specialized outpatient care) and Cause of Death Register were used to collect information concerning alcohol-related morbidity and mortality ICD-10 codes (from 1997). The Swedish index of alcohol-related diagnoses was used, which includes diagnoses such as alcoholic psychoses, alcohol use disorder, alcoholic liver disease, and toxic effect of alcohol (26). From the Social Insurance Agency’s MiDAS register, information on alcohol-related sickness absence and disability pension was included using the ICD-10 codes related to mental and behavioral disorders due to alcohol (F10), alcoholic liver disease (K70), and toxic effect of alcohol (T51). Lastly, to capture individuals visiting primary care for their alcohol use disorder (27), we included information from the National Prescribed Drug Register to identify cases with prescriptions for alcohol use disorders based on the following anatomical therapeutic chemical (ATC) classification disulfiram (N07BB01), acamprosate (N07BB03), naltrexone (N07BB04), and nalmefene (N07BB05) (28).

In the analyses, a composite measure of alcohol-related health problems was created, combining information from the four different registers mentioned above. Thus, the outcome includes a range of indicators, from a single prescription for alcohol dependence medication to alcohol-related mortality. The first incidence of diagnoses, either as principal or contributory diagnoses, or prescribed medication were identified.

### Covariates

Covariate selection was informed by previous research (14). From the LISA register, we obtained baseline data on sex, age (continuous), highest level of education (primary ≤9, secondary 10–12, university >12 years of schooling), country of birth (Sweden or outside of Sweden), civil status (single, married/in partnership) and presence of children <18 years in the household (yes, no). Parental education (primary, secondary, university) was obtained by linking participants to their parents via the MGR, capturing early-life socioeconomic context. These demographic factors may influence occupational status and exposures, help-seeking behavior, and alcohol-related outcomes. Furthermore, country of birth may influence cultural norms around alcohol; having a partner can provide social support buffering against job strain, while children may increase stress but reduce problem drinking.

Participant mental health diagnoses (ICD-10: F00–F99, plus equivalent ICD-9/8 codes) requiring inpatient care prior to 2005 were retrieved from the National Patient Register. Parental mental health and alcohol-related morbidity/mortality were obtained from the National Patient Register and the Cause of Death Register, using definitions described earlier. These health-related variables account for pre-existing vulnerability and familial predisposition, which may confound associations between job strain and alcohol-related outcomes.

### Statistical analysis

Pearson’s chi-square tests (χ2) were used to test the descriptive differences of the study population. The associations between job strain and social support and alcohol‐related health problems were estimated using Cox’s proportional hazard models to obtain hazard ratios (HR) with 95% confidence intervals (CI). The proportional hazards assumption was assessed using Schoenfeld residuals and log-log plots; although statistical tests indicated minor violations, visual inspection suggested the assumption was reasonably met. Person-time was calculated from 1 January 2006 until the date of alcohol-related diagnoses or drug prescription, date of emigration, date of death, or end of follow-up (31 December 2020), whichever came first.

To assess the moderating role of social support on the association between job strain and alcohol-related health problems, we calculated the relative excess risk due to the interactions (RERI) [RERI=HR_11_-HR_10_-HR_01_+1]. RERI was used to assess the presence of an additive interaction following recommendations for Cox regression models (29). The 95% CI was estimated according to the Delta method models. The attributable proportion (AP) due to interaction was also calculated (AP=RERI/HR_11_). Effect modification was evaluated using the additive scale as it is recommended for assessing the public health significance of interactions (26). In line with recommendations, we created a joint exposure variable based on job strain and workplace social support to ensure our interaction analyses have a single common reference group (30). In the main analysis, the reference category was low-strain jobs with strong workplace social support.

Given that previous research suggests there could be sex differences in the vulnerability to the effects of job strain and alcohol-related health problems (14, 17, 20), we tested for multiplicative interaction between sex and the occupational exposures in relation to alcohol-related health problems.

First, a crude model was fit. The second model was adjusted for age, country of birth, the highest level of education, and prior mental health problems. In the third model, additional adjustments for parents’ highest level of education, and parents’ mental and alcohol-related health problems were made.

Missing values on covariates were coded as separate categories as similar results were reached when doing complete case analyses (supplementary tables S3 and S4). An additional analysis stratified on level of education was conducted to examine if associations and roles of workplace social support differ depending on the level of education. This was done as we are unable to investigate differences in occupation, sector, or socioeconomic class as these factors are strongly correlated to the JEM. All analyses were performed using Stata Statistical Software, release 17, (StataCorp, College Station, TX, USA).

## Results

The baseline characteristics, stratified by job strain, are found in table 1. In general, more women had high-strain jobs compared to all other groups of job strain. A larger proportion of individuals born outside of Sweden had either a high-strain or passive job. Individuals in active jobs were generally married with children under the age of 18. Individuals in passive jobs were generally less educated and had less educated parents, as well as a higher prevalence of parents with alcohol-related health problems. Baseline characteristics stratified by job strain and sex can be found in supplementary table S5.

**Table 1:** Baseline characteristics of the study population, stratified by job strain. [SD=standard deviation]

		Active			High strain			Low strain			Passive			P-value
		N (%)	Mean (SD)		N (%)	Mean (SD)		N (%)	Mean (SD)		N (%)	Mean (SD)		
Total		990 970 (35.1)			507 829 (18.0)			366 444 (13.0)			957 219 (33.9)			
Sex														
	Male	992 304 (49.7)			209 899 (41.3)			207 207 (56.6)			514 138 (53.7)			<0.001
	Female	498 666 (50.3)			297 930 (58.7)			159 237 (43.5)			443 081 (46.3)			
Age			44.8 (8.9)			44.7 (8.8)			44.0 (8.9)			44.2 (8.9)		
Country of birth														
	Swedish	907 788 (91.2)			439 559 (86.6)			334 391 (91.3)			801 798 (83.8)			<0.001
	Non-Swedish	85 152 (8.4)			68 220 (13.4)			32 036 (8.7)			155 176 (16.2)			
	Missing	30 (0.0)			50 (0.0)			17 (0.0)			245 (0.0)			
Civil status														
	Single	299 558 (30.2)			179 976 (35.4)			127 706 (34.9)			365 891 (38.2)			<0.001
	Married/ in partnership	691 412 (69.8)			327 853 (64.6)			238 738 (65.2)			591 328 (61.8)			
Presence of children <18 years in the household														
	Yes	507 831 (51.3)			248 610 (49.0)			177 767 (48.5)			446 752 (46.7)			<0.001
	No	483 139 (48.8)			259 219 (51.0)			188 677 (51.5)			510 467 (53.3)			
Highest level of education														
	Primary	52 614 (5.3)			55 118 (10.9)			31 019 (8.5)			217 490 (22.7)			<0.001
	Secondary	296 363 (29.9)			270 768 (53.3)			185 567 (50.6)			621 391 (64.9)			
	University	640 283 (64.6)			181 037 (35.7)			149 289 (40.7)			115 723 (12.1)			
	Missing	1710 (0.2)			906 (0.2)			569 (0.2)			2615 (0.3)			
Prior own mental health problems		34 781 (3.5)			26 720 (5.3)			14 323 (3.9)			52 027 (5.5)			<0.001
Parents highest level of education														
	Primary	269 869 (27.3)			179 358 (35.3)			116 899 (31.9)			377 149 (39.4)			<0.001
	Secondary	343 175 (34.6)			173 410 (34.2)			132 753 (36.2)			312 933 (32.7)			
	University	263 857 (26.6)			73 972 (14.6)			75 248 (20.5)			89 737 (9.4)			
	Missing	114 069 (11.5)			81 089 (16.0)			41 544 (11.3)			177 400 (18.5)			
Parents alcohol-related health problems		51 722 (5.2)			30 037 (5.9)			20 943 (5.7)			62 104 (6.5)			<0.001
Parents mental health problems		242 475 (24.5)			127 563 (25.1)			92 266 (25.2)			245 104 (25.6)			<0.001

During follow-up, a total of 94 900 (3.4%) received care, a prescription or died because of an alcohol-related diagnoses, of which drug prescription (41.6%) and in- and outpatient care (24.1% and 31.8%, respectively) were most common for alcohol-related health problems (supplementary table S6). A total of 64 235 (67.7%) men and 30 665 (32.3%) women were defined as having the outcome of interest. The average follow-up time for men and women was 13.8 years and 14.2 years, respectively.

### Main analyses

Multiplicative interaction terms were significant for job strain (P<0.001), workplace social support (P=0.02), and the combined exposure of job strain and workplace social support (P<0.001). Thus, the main analyses were stratified by sex. The main effect of job strain and social support, stratified by sex, are shown in table 2. Compared to individuals in low-strain jobs, men in both high-strain and passive jobs were found to have an increased risk of alcohol-related health problems in the crude model. The initial elevated risks remained, slightly attenuated for men in high strain (HR 1.28, 95% CI 1.25–1.32) and passive jobs (HR 1.32, 95% CI 1.28–1.35) after adjusting for individual and parental covariates. For women, in the crude model, active jobs were protective against alcohol-related health problems, while high-strain and passive jobs increased the risk. The initial elevated risks remained for women in high-strain (HR 1.06, 95% CI 1.01–1.10) and passive jobs (HR 1.05, 95% CI 1.01–1.09) in the final model.

**Table 2:** Crude and adjusted hazard ratios (HR) with 95% confidence intervals (CI) for the association of job strain and social support on the outcome of alcohol-related health problems, stratified by sex.

			Number of events		Model 1 ^a^		Model 2 ^b^		Model 3 ^c^
			N (%)		HR (95% CI)		HR (95% CI)		HR (95% CI)
Men									
	Job strain								
		Active	17 491 (3.6)		1.03 (1.00–1.06)		1.07 (1.05–1.11)		1.07 (1.04–1.10)
		High strain	10 603 (5.1)		1.47 (1.43–1.51)		1.29 (1.25–1.33)		1.28 (1.25–1.32)
		Low strain (reference)	7221 (3.5)		1.00		1.00		1.00
		Passive	28 920 (5.6)		1.64 (1.60–1.68)		1.32 (1.29–1.36)		1.32 (1.28–1.35)
	Social support								
		Strong social support (reference)	30 886 (4.4)		1.00		1.00		1.00
		Weak social support	33 349 (4.7)		0.94 (0.93–0.96)		0.96 (0.94–0.97)		0.96 (0.94–0.97)
Women									
	Job strain								
		Active	8410 (1.7)		0.75 (0.72–0.78)		0.89 (0.86–0.93)		0.89 (0.85–0.92)
		High strain	7038 (2.4)		1.05 (1.00–1.09)		1.06 (1.02–1.10)		1.06 (1.01–1.10)
		Low strain (ref)	3594 (2.3)		1.00		1.00		1.00
		Passive	11 623 (2.6)		1.17 (1.12–1.21)		1.04 (1.00–1.08)		1.05 (1.01–1.09)
	Social support								
		Strong social support (reference)	15 631 (2.1)		1.00		1.00		1.00
		Weak social support	15 034 (2.3)		0.95 (0.93–0.97)		1.08 (1.06–1.11)		1.08 (1.06–1.11)

^a^ Model 1: Crude. ^b^ Model 2: Adjusted for sex, age, country of birth, civil status, presence of children under 18 years in the household, the highest level of education, and prior mental health problems. ^c^ Model 3: Additional adjustments for parents’ highest level of education, and parents’ mental and alcohol-related health problems.

Compared to strong workplace social support, weak workplace social support was protective for both men (HR 0.94, 95% CI 0.93–0.96) and women (HR 0.95, 95% CI 0.93–0.97) in relation to alcohol-related health problems in the crude model. The strength and direction of the association remained for men in the fully adjusted model. However, for women with weak workplace social support, compared to strong social support, an increased risk of alcohol-related health problems was found in the final model (HR 1.08, 95% CI 1.06–1.11).

### Effect modification of workplace social support

Among men, a negative interaction between job strain and levels of workplace social support on alcohol-related health problems was identified when compared to men with low-strain jobs and high levels of workplace social support (table 3). In the final model, a negative interaction effect was observed for individuals in high-strain and passive jobs (AP -0.37 and -0.06, respectively), suggesting that the combined effect of job strain and low workplace social support was smaller than expected based on their individual effects. After full adjustments, men in high-strain and passive jobs had an increased risk of alcohol-related health problems irrespective of level of workplace social support.

**Table 3:** Crude and adjusted hazard ratios (HR) with 95% confidence intervals (CI) for the association of job strain combined with social support on the outcome of alcohol-related health problems, stratified by sex. [RERI=relative risk due to interaction; AP=attributable proportion]

			Number of events		Model 1 ^a^		Model 2 ^b^		Model 3 ^c^	RERI ^d^ (95% CI)	AP ^e^ (95% CI)
			N (%)		HR (95% CI)		HR (95% CI)		HR (95% CI)		
Men											
	Active										
		Strong level of support	6673 (3.5)		1.13 (1.09–1.18)		1.08 (1.03–1.12)		1.07 (1.03–1.12)		
		Weak level of support	10 818 (3.6)		1.18 (1.13–1.22)		1.13 (1.09–1.17)		1.13 (1.09–1.17)	-0.02(-0.08–0.03)	-0.01 (-0.7–0.4)
	High strain										
		Strong level of support	3268 (5.9)		1.96 (1.87–2.05)		1.61 (1.53–1.69)		1.61 (1.54–1.69)		
		Weak level of support	7335 (4.8)		1.55 (1.49–1.61)		1.23 (1.18–1.28)		1.23 (1.18–1.28)	-0.45 (-0.53– -0.36)	-0.37 (-0.43– -0.30)
	Low strain										
		Strong level of support (ref)	3819 (3.1)		1.00		1.00		1.00		
		Weak level of support	3402 (4.1)		1.32 (1.26–1.38)		1.06 (1.01–1.11)		1.07 (1.02–1.12)		
	Passive										
		Strong level of support	19 589 (5.6)		1.85 (1.79–1.92)		1.37 (1.32–1.42)		1.37 (1.31–1.42)		
		Weak level of support	9331 (5.6)		1.84 (1.77–1.91)		1.35 (1.30–1.40)		1.35 (1.30–1.41)	-0.09 (-0.15– -0.02)	-0.06 (-0.11– -0.02)
Women											
	Active										
		Strong level of support	2314 (1.9		0.92 (0.86–0.98)		0.97 (0.91–1.04)		0.96 (0.90–1.02)		
		Weak level of support	6096 (1.6)		0.80 (0.76–0.84)		0.96 (0.91–1.01)		0.95 (0.90–1.01)	-0.16 (-0.25– -0.07)	-0.16 (-0.26–- -0.08)
	High strain										
		Strong level of support	4655 (2.5)		1.23 (1.16–1.30)		1.13 (1.06–1.19)		1.12 (1.06–1.18)		
		Weak level of support	2383 (2.1)		1.05 (0.99–1.12)		1.18 (1.11–1.26)		1.17 (1.10–1.25)	-0.10 (-0.20– -0.01)	-0.09 (-0.17– -.01)
	Low strain										
		Strong level of support (ref)	1679 (2.0)		1.00		1.00		1.00		
		Weak level of support	1915 (2.5)		1.22 (1.15–1.31)		1.16 (1.09–1.24)		1.15 (1.08–1.23)		
	Passive										
		Strong level of support	6386 (2.3)		1.15 (1.09–1.21)		1.03 (0.98–1.09)		1.03 (0.98–1.09)		
		Weak level of support	5237 (3.1)		1.53 (1.45–1.61)		1.26 (1.19–1.33)		1.26 (1.19–1.33)	0.08 (-0.01–0.16)	0.06 (-0.00–0.12)

^a^ Model 1: Crude. ^b^ Model 2: Adjusted for age, country of birth, civil status, presence of children under 18 years in the household, the highest level of education, and prior mental health problems. ^c^ Model 3: Additional adjustments for childhood socioeconomic position, parents’ highest level of education, and parents’ mental and alcohol-related health problems. ^d^ HR_11_-HR_10_-HR_01_+1. ^e^ RERI/HR_11_.

In the fully adjusted model, women in high-strain jobs had an increased risk of alcohol-related health problems, regardless of their level of workplace social support, compared to women in low-strain jobs with strong workplace social support (table 3). Similar to the results for males, a negative interaction was observed between high strain and weak workplace social support (AP -0.09). Women in passive jobs with weak levels of workplace social support were at an increased risk of alcohol-related health problems (HR 1.26, 95% CI 1.19–1.33), with an AP of 6, implying that around 6% of the excess risk among women in passive jobs is attributable to the interaction between job strain and workplace social support.

### Additional analyses

Results further stratified on level of education are presented in supplementary tables S7 and S8. Among men, high-strain and passive jobs increases the risk of alcohol-related health problems irrespective of education level and workplace social support. Similar patterns of results were found among women with secondary or university level of education. Among women with high education, there was a more pronounced increase in the risk of alcohol-related health problems when they have passive jobs with weak levels of workplace social support.

## Discussion

The current study aimed to examine the association between job strain and alcohol-related health problems among men and women and the extent to which workplace social support moderates this association. Results indicate that job strain and workplace social support are associated with alcohol-related health problems among both men and women, with varying degree and direction. Among both men and women, high-strain and passive jobs were associated with an increased risk of alcohol-related health problems. This risk was more pronounced among men. Workplace social support appears to slightly modify the association between job strain and alcohol-related health problems, however, in different directions and magnitudes depending on the psychosocial exposure and sex of the worker.

The current results further strengthen and extend existing research on the association between job strain and alcohol-related health problems. Previous research has found an increased risk of alcohol-related morbidity among men in high-strain and passive jobs as well as for women in passive jobs (14, 15). This was also found in the current study as well as an increased risk among women in high-strain jobs. This is important because high-strain jobs are very common among women (eg, nursing, teaching, retail work, administrative assistance). Consequently, the stress from these roles may lead to increased consumption of alcohol as a coping mechanism, thereby heightening the risk of alcohol-related health problems. In the current study, we included a broader measure of alcohol-related health problems. Using multiple sources of potential treatment is more representative and captures more cases/ avoids misclassification of the outcome that may have been present in the previous study (14). In Sweden, sickness absence and disability pension are more common among women compared to men (31). The commonality among high-strain and passive jobs is the dimension of low job control, which appears to be more important compared to high demands in relation to the outcome of alcohol-related health problems which have been shown in previous research (14). A potential reason for this could be those with low job control, little autonomy, or limited decision-making power may experience alienation from work (32). This, in turn, could lead to increased alcohol consumption to cope with the feeling of boredom (33).

In line with previous research, we found that the strength of the association between job strain and alcohol-related health problems was stronger for men compared to women (14, 20). Evidence suggests that men and women are equally sensitive to the psychosocial exposures at work (34). Consequently, the risk differences in the associations between job strain and alcohol-related health problems could be due to several different reasons. For example, women tend to consume less alcohol compared to men, in part due to biological differences in alcohol metabolism, and have less alcohol-related health problems (35). Also, the Swedish labor market is gendered (36), and, subsequently, men and women are exposed differently as they work in different occupations and have different tasks and exposures to job strain and workplace social support (22, 24). The alcohol norms and habits of these different occupations tend to vary, which could also partially explain the current results (6, 7, 37). Furthermore, men and women's coping skills when dealing with work-related stress differ. Men tend to externalize distress and are more likely to increase their drinking in response to work stressors compared to women (10).

We found varying effects of workplace social support among men and women, which is in line with previous research (17, 19, 20). Contradicting previous research, we found that men with strong workplace social support had an increased risk of alcohol-related health problems (15, 20). Among women, however, weak workplace social support increased the risk of alcohol-related health problems. This supports the notion that different employees might require different forms of social support depending on different work stressors and that social support can have different effects on alcohol consumption (19). Previous research has challenged the belief that strong workplace social support reduces alcohol consumption as it may foster a greater sense of community, potentially increasing drinking (38). Furthermore, there could be differences between men and women regarding how social support is perceived and to what extent social support is sought (39). In the current study, social support information is aggregated at the occupational level, combining support from both colleagues and supervisors. This measure may differ from self-reported information on social support.

Regarding our second aim, we did not find substantial evidence that workplace social support modifies the association between job strain and alcohol-related health problems. This finding aligns with previous research on self-reported alcohol consumption (19). For men, the association between both high-strain and passive jobs and the outcome of alcohol-related health problems was strong irrespective of the level of workplace social support. While for women, we saw that the association between job strain and alcohol-related health problems was only modified by strong levels of workplace social support among women in passive jobs. In this study, we examined general workplace social support rather than specific dimensions that may explain the findings. Prior research suggests that support dimensions like tangible belonging, and appraisal support can influence the relationship between work stress and alcohol use (19). Our aggregated support measure encompassed emotional, instrumental, and appraisal support, but may have lacked the specificity needed to detect buffering effects on job strain. Future research should investigate which aspects of workplace social support and types of work stress interact to influence alcohol-related harm.

### Strengths and limitations

The strengths of the current study include the use of register-based data to follow a large cohort of the Swedish working population over an extended period. The exposure and outcome were well-defined, and information was obtained from registers, minimizing the risk of attrition and self-report bias. Another strength of using the JEM to measure job strain and workplace social support is that the perceived job strain and support could systematically differ between individuals with and without alcohol-use problems. However, using the JEM to measure exposure to psychosocial work environments has some limitations as this information is aggregated at the occupational level and only measured at a single time-point. Measuring the work environment exposure at the aggregated level could lead to some misclassification bias as there could be large variation in levels of job demands, job control, and social support within the same occupation, and it is difficult to capture subjective psychosocial factors via aggregated measures. Therefore, while our results provide insights into general patterns, future studies using individual-level data are needed to complement these findings. Using only a single measurement point for exposure may introduce misclassification bias as individuals can change occupations and, consequently, their exposure levels. However, a previous study using the same cohort indicated that psychosocial work environment exposures on the occupational level tend to remain stable over time (24). Differential misclassification may have occurred if individuals with alcohol-related health problems change jobs prior to receiving a formal diagnosis. For example, someone struggling with alcohol use might leave a high-strain job due to declining health or performance and only later receive a diagnosis. This could lead to underestimation of the association between job strain and alcohol-related health outcomes. Although we extended previous research by including several registers to define varying degrees of alcohol-related health problems, using a composite outcome that varies in severity may obscure differences in risk factors and pathways leading to distinct alcohol-related health problems. Future research should consider stratifying outcomes by severity or type to better capture these differential associations. There is a risk of unmeasured confounding from, for example, individual lifestyle factors and work schedule. Lastly, the generalizability of the current results to other contexts outside of Sweden could be limited due to differences in alcohol policy and work-related context.

### Concluding remarks

In Sweden, job strain is associated with an increased risk of alcohol-related health problems, particularly among men. Women, however, appear to experience the protective effects of strong work-related social support measured at the occupational level. Future research is needed to fully understand the role of work-related social support in alcohol-related harm among both men and women and how different types of social support may have varying effects.

## Supplementary material

Supplementary material
