# Supplementary material for: Job strain, social support, and alcohol-related health problems: A register-based cohort study
Source: Scand J Work Environ Health. 2025 Dec 27;52(1):31–40. doi: 10.5271/sjweh.4257 (PMC12776427; doi:10.5271/sjweh.4257)
Supplement: Supplementary material [file SJWEH-52-31-S001.pdf]

Job strain, social support, and alcohol-related health problems: A register-based cohort study[1]

by Emelie Thern, PhD,[2] Erica Jonsson, MSc, Devy L Elling, PhD, Melody Almroth, PhD[1]

1. Supplementary material
2. Correspondence to: Emelie Thern, Unit of Occupational Medicine, Karolinska Institutet, Solnavägen 4, 113 65 Stockholm. [E-mail: emelie.thern@ki.se]

Supplementary Table S1: Baseline characteristics of included and excluded individuals

	Included<br>n(%)	Excluded<br>n(%)	p-value
Total	2 882 462 (74.1)	984 486 (25.9)	
Sex			
Male	1 426 548 (50.4)	506 589 (51.5)	<0.001
Female	1 398 914 (49.6)	477 897 (48.5)	
Age			
Mean ± SD	44.5 ± 8.9	46.3±9.2	
Country of birth			
Swedish	2 483 536 (88.0)	714 444 (72.6)	<0.001
Non-Swedish	338 584 (12.0)	268 791 (27.3)	
Missing	342 (0.0)	1251 (0.1)	
Civil status			
Single	973 131 (34.5)	472 375 (48.0)	<0.001
Married/ in partnership	1 849 331 (65.5)	512 111 (52.0)	
Presence of children under 18 years in the household			
Yes	1 380 960 (48.9)	342 261 (34.8)	<0.001

No	1 441 502 (51.1)	642 225 (65.2)	
Highest level of education			
Primary	356 241 (12.6)	253 712 (25.8)	<0.001
Secondary	1 374 089 (48.7)	469 983 (47.7)	
University	1 086 332 (38.5)	228 801 (23.2)	
Missing	5800 (0.2)	31 990 (3.3)	
Prior own mental health problems	128 760 (4.6)	212 620 (21.6)	<0.001
Parents highest level of education			
Primary	943 275 (33.4)	317 201 (32.2)	<0.001
Secondary	962 271 (34.1)	254 654 (25.9)	
University	502 814 (17.8)	108 623 (11.0)	
Missing	414 102 (14.7)	304 008 (30.9)	
Parents alcohol-related health problems	164 806 (5.8)	57 553 (5.9)	0.801
Parents mental health problems	707 408 (25.1)	235 334 (23.9)	<0.001

Supplementary Table S2: Questions included in the dimensions of job control, job demands and social support

Dimension	Question	Answer alternatives
Job control		
Decision authority	Can you partially decide when tasks should be done?	Never, mostly not, mostly, always
	Do you have the opportunity to decide your own work pace?	Not at all, occasionally, roughly ¼ of the time, half of the time, roughly ¾ of the time, almost all the time
	Can you take short breaks to talk pretty much any time?	Not at all, occasionally, roughly ¼ of the time, half of the time, roughly ¾ of the time, almost all the time
	Are you ever involved in deciding how your work is organized?	Never, mostly not, mostly, always
Skill discretion	Is there any apprenticeship or introductory training required at the workplace (besides education or course) before you can do your job?	No, only a few days, a few weeks, a few months, ½ year, 1 years, 2 years, 3 years, 4 years or more
	Does the work require you to repeat the same work steps many times an hour?	Not at all, occasionally, roughly ¼ of the time, half of the time, roughly ¾ of the time, almost all the time
	Do you spend quite some time during the day trying to understand or solving difficult problems?	Not at all, occasionally, roughly ¼ of the time, half of the time, roughly ¾ of the time, almost all the time
	Does the work offer you the possibility to learn something new and to develop in this occupation?	Not at all, a few days per month, one day per week, a few days per week, every day.
Job demands		
Psychological demands	Are you sometimes so stressed that you do not have time to talk about or even think about something besides work?	Not at all, occasionally, roughly ¼ of the time, half of the time, roughly ¾ of the time, almost all the time
	Do you sometimes have so much to do that you have to work during lunch, work overtime, or take work home?	Not at all, a few days per month, one day per week, a few days per week, every day.

	Does your work require all your attention and concentration?	Not at all, occasionally, roughly ¼ of the time, half of the time, roughly ¾ of the time, almost all the time
Workplace social support		
	Do you have the opportunity to receive support and encouragement from colleagues when work feels difficult?	Never, mostly not, mostly, always
	Do you have the opportunity to receive support and encouragement from managers when work feels difficult?	Never, mostly not, mostly, always
	If the tasks feel too difficult, do you have the opportunity to get advice or help?	Never, mostly not, mostly, always
	Does your manager show appreciation for something you have done?	Not at all, a few days per month, one day per week, a few days per week, every day.
	Do other people show appreciation for something you have done?	Not at all, a few days per month, one day per week, a few days per week, every day.

Supplementary Table S3. Complete case analysis excluding 415 198 individuals with missing information on covariates: crude and adjusted hazard ratios (HR) with 95% confidence intervals (CI) for alcohol-related health problems by job stain and social support

	Number of events n (%)	Model 1 (95% CI)	Model 2 (95% CI)	Model 3 (95% CI)
**Men**				
Job strain				
Active	15 485 (3.6)	1.03 (1.00- 1.06)	1.08 (1.05- 1.11)	1.07 (1.04- 1.11)
High strain	9073 (5.3)	1.54 (1.49- 1.59)	1.33 (1.28- 1.37)	1.32 (1.28- 1.36)
Low strain (ref)	6446 (3.5)	1.00	1.00	1.00
Passive	24 813 (5.9)	1.71 (1.66- 1.75)	1.35 (1.32- 1.39)	1.35 (1.31- 1.39)
Social support				
Strong social support (ref)	26 832 (4.4)	1.00	1.00	1.00
Weak social support	28 985 (4.7)	0.94 (0.93- 0.96)	0.95 (0.94- 0.97)	0.96 (0.94- 0.97)
**Women**				
Job strain				

Active	7302 (1.7)	0.74 (0.71- 0.77)	0.89 (0.86- 0.93)	0.89 (0.85- 0.32)
High strain	6079 (2.4)	1.07 (1.03- 1.12)	1.09 (1.04- 1.14)	1.09 (1.04- 1.13)
Low strain (ref)	3093 (2.2)	1.00	1.00	1.00
Passive	9829 (2.8)	1.25 (1.20- 1.30)	1.09 (1.04- 1.13)	1.09 (1.05- 1.14)
Social support				
Strong social support (ref)	13 261 (2.2)	1.00	1.00	1.00
Weak social support	13 042 (2.3)	0.94 (0.92- 0.96)	1.09 (1.07- 1.11)	1.09 (1.06- 1.12)

Model 1: crude

Model 2: adjusted for sex, age, country of birth, civil status, presence of children under 18 years in the household, the highest level of education, and prior mental health problems

Model 3: additional adjustments for parents' highest level of education, and parents' mental and alcohol-related health problems

Supplementary Table S4. Complete case analysis excluding 415 198 individuals with missing information on covariates: crude and adjusted hazard ratios (HR) with 95% confidence intervals (CI) for the association of job strain and social support on the outcome of alcohol-related health problems, stratified by sex.

	Number of events n (%)	Model 1 (95% CI)	Model 2 (95% CI)	Model 3 (95% CI)	RERI (95% CI)	AP
**Men**						
Active						
Strong level of support	5998 (3.5)	1.13 (1.08- 1.18)	1.07 (1.04- 1.12)	1.07 (1.03- 1.12)		
Weak level of support	9487 (3.6)	1.18 (1.14- 1.23)	1.13 (1.08- 1.17)	1.12 (1.08- 1.17)	-0.01 (-0.07- 0.05)	-0.01 (-0.06- 0.05)
High strain						
Strong level of support	2672 (6.3)	2.09 (1.99- 2.20)	1.68 (1.60- 1.77)	1.66 (1.58- 1.75)		
Weak level of support	6401 (4.9)	1.61 (1.54- 1.68)	1.26 (1.24- 1.31)	1.26 (1.20- 1.31)	-0.47 (-0.56- -0.37)	-0.37 (-0.44- -0.30)

Low strain						
Strong level of support (ref)	3444 (3.1)	1.00	1.00	1.00		
Weak level of support	3002 (4.0)	1.31 (1.25- 1.37)	1.05 (1.00- 1.11)	1.06 (1.01- 1.12)		
Passive						
Strong level of support	16 871 (5.9)	1.93 (1.86- 2.00	1.40 (1.34- 1.48)	1.39 (1.34- 1.46)		
Weak level of support	7942 (5.8)	1.90 (1.82- 1.98)	1.37 (1.31- 1.43)	1.37 (1.31- 1.43)	-0.08 (-0.15- -0.02)	-0.06 (0.11 - - 0.01)
**Women**						
Active						
Strong level of support	2038 (1.8)	0.91 (0.85- 0.97)	0.99 (0.91- 1.04)	0.96 (0.89- 1.02)		
Weak level of support	5264 (1.6)	0.79 (0.75- 0.84)	0.96 (0.90- 1.02)	0.96 (0.90- 1.01)	-0.15 (-0.24- -0.05)	-0.16 (-0.25- - 0.06)
High strain						
Strong level of support	4021 (2.6)	1.26 (1.19- 1.34)	1.16 (1.09- 1.23)	1.15 (1.08- 1.22)		
Weak level of support	2058 (2.2)	1.06 (0.99- 1.14)	1.20 (1.12- 1.29)	1.20 (1.12- 1.28)	-0.10 (-0.20- 0.00)	-0.08 (-0.17- 0.00)

Low strain						
Strong level of support (ref)	1451 (2.0)	1.00	1.00	1.00		
Weak level of support	1642 (2.5)	1.23 (1.14- 1.32)	1.16 (1.08- 1.24)	1.14 (1.07- 1.23)		
Passive						
Strong level of support	5532 (2.4)	1.20 (1.14- 1.28)	1.06 (1.00- 1.13)	1.06 (1.00- 1.13)		
Weak level of support	4297 (3.4)	1.71 (1.61- 1.81)	1.35 (1.27- 1.44)	1.34 (1.26- 1.42)	0.13 (0.05- 0.22)	0.10 (0.03 – 0.17)

Relative risk due to interaction (RERI) = $HR_{11} - HR_{10} - HR_{01} + 1$.

Attributable proportion (AP) = $RERI/HR_{11}$

Model 1: Crude

Model 2: Adjusted for age, country of birth, civil status, presence of children under 18 years in the household, the highest level of education, and prior mental health problems

Model 3: Additional adjustments for childhood socioeconomic position, parents' highest level of education, and parents' mental and alcohol-related health problems

Supplementary Table S5. Baseline characteristics of the study population, stratified by sex job strain.

	Active n(%)	High strain n(%)	Low strain n(%)	Passive n(%)	p-value
**Men**					
Total	504 619 (33.9)	221 503 (14.9)	213 200 (14.3)	550 099 (36.9)	
Age					
Mean ± SD	45.4 ± 8.9	44.5 ± 8.9	43.4 ± 8.8	44.0 ± 8.8	
Country of birth					
Swedish	454 291 (92.3)	177 820 (84.7)	190 611 (92.0)	437 318 (85.1)	<0.001
Non-Swedish	37 999 (7.7)	32 040 (15.3)	16 587 (8.0)	76 669 (14.9)	
Missing	14 (0.0)	39 (0.0)	9 (0.0)	151 (0.0)	
Civil status					
Single	140 801 (28.6)	81 775 (39.0)	72 846 (35.2)	210 092 (40.9)	<0.001
Married/ in partnership	351 503 (71.4)	128 124 (61.0)	135 361 (64.8)	304 046 (59.1)	
Presence of children under 18 years in the household					

Yes	242 058 (49.2)	91 092 (43.4)	98 424 (47.5)	218 816 (42.6)	<0.001
No	250 246 (50.8)	91 092 (43.4)	98 424 (47.5)	218 816 (42.6)	
Highest level of education					
Primary	37 093 (7.5)	41 265 (19.7)	17 388 (8.4)	122 271 (23.8)	<0.001
Secondary	181 562 (36.9)	98 748 (47.1)	93 945 (45.3)	331 770 (64.5)	
University	272 402 (55.3)	69 191 (33.0)	95 434 (46.1)	58 620 (11.4)	
Missing	1247 (0.3)	695 (0.3)	440 (0.2)	1477 (0.3)	
Prior own mental health problems	13 576 (2.8)	9974 (4.8)	6348 (3.1)	26 387 (5.1)	<0.001
Parents highest level of education					
Primary	139 674 (28.4)	71 853 (34.2)	62 516 (30.2)	204 036 (39.7)	<0.001
Secondary	170 507 (34.6)	68 170 (32.5)	76 015 (36.7)	172 383 (33.5)	
University	126 375 (25.7)	32 886 (15.7)	47 868 (23.1)	49 868 (9.7)	
Missing	55 748 (11.3)	36 990 (17.6)	20 808 (10.0)	87 851 (17.1)	
Parents alcohol-related health problems	25 140 (5.1)	12 241 (5.8)	11 549 (5.6)	33 256 (6.5)	<0.001
Parents mental health problems	120 856 (24.6)	52 007 (24.8)	51 782 (25.0)	133 509 (26.0)	<0.001
**Women**					

Total	526 803 (34.4)	325 708 (21.3)	172 738 (11.3)	504 789 (33.0)	
Age					
Mean ± SD	44.3 ±8.9	44.9 ± 8.6	44.8 ± 8.9	44.4 ± 8.9	
Country of birth					
Swedish	453 497 (90.9)	261 739 (87.9)	143 780 (90.3)	364 480 (82.3)	<0.001
Non-Swedish	45 153 (9.1)	36 180 (12.1)	15 499 (9.7)	78 507 (17.7)	
Missing	16 (0.0)	11 (0.0)	8 (0.0)	94 (0.0)	
Civil status					
Single	158 757 (31.8)	98 201 (33.0)	54 860 (34.5)	155 799 (35.2)	<0.001
Married/ in partnership	339 909 (68.2)	199 720 (67.0)	104 377 (65.6)	2847 282 (64.8)	
Presence of children under 18 years in the household					
Yes	265 773 (53.3)	157 518 (52.9)	79 343 (49.8)	227 936 (51.4)	<0.001
No	232 893 (46.7)	140 412 (47.1)	79 894 (50.2)	215 145 (48.6)	
Highest level of education					
Primary	15 521 (3.1)	13 853 (4.7)	13 631 (8.6)	95 219 (21.5)	<0.001

Secondary	114 801 (23.0)	172 020 (57.7)	91 622 (57.5)	289 621 (65.4)	
University	367 881 (73.8)	111 846 (37.5)	53 855 (33.8)	57 103 (12.9)	
Missing	463 (0.1)	211 (0.1)	120 (0.1)	1138 (0.3)	
Prior own mental health problems	21 205 (4.3)	16 746 (5.6)	7884 (5.0)	26 640 (6.0)	<0.001
Parents highest level of education					
Primary	130 195 (26.1)	107 505 (36.1)	54 383 (34.2)	173 113 (39.1)	<0.001
Secondary	172 668 (34.6)	105 240 (35.3)	56 738 (35.6)	140 550 (31.7)	
University	137 482 (25.6)	41 086 (13.8)	27 380 (17.2)	39 869 (9.0)	
Missing	58 321 (11.7)	44 099 (14.8)	20 736 (13.0)	89 549 (20.2)	
Parents alcohol-related health problems	26 582 (5.3)	17 796 (6.0)	9394 (5.9)	28 848 (6.5)	<0.001
Parents mental health problems	121 619 (24.4)	75 556 (25.4)	40 484 (25.4)	111 595 (25.2)	<0.001

SD: Standard deviation

Supplementary Table S6: Prevalence of each component included in the composite outcome score of alcohol-related health problems

Alcohol dimension	n(%)
Alcohol-related inpatient care	22 859 (24.1)
Alcohol-related outpatient care	30 208 (31.8)
Alcohol-related cause of death	2190 (2.3)
Alcohol-related sickness absence and disability pension	137 (0.0)
Drug prescription for alcohol use disorder	39 506 (41.6)
Alcohol-related health problems (total)	94 900

Supplementary Table S7. Crude and adjusted hazard ratios (HR) with 95% confidence intervals (CI) for the association of job strain and social support on the outcome of alcohol-related health problems for *men*, stratified by education

	Number of events n(%)	Crude (95% CI)	Adjusted (95% CI)	RERI (95% CI)	AP
**Primary**					
Active					
Strong level of support	866 (6.2)	1.02 (0.89- 1.16)	1.08 (0.95- 1.24)		
Weak level of support	1477 (5.9)	0.96 (0.85- 1.09)	1.06 (0.94- 1.21)	-0.11 (-0.30- 0.06)	-0.11 (-0.27 – 0.05)
High strain					
Strong level of support	760 (8.3)	1.35 (1.17- 1.55)	1.36 (1.18- 1.57)		
Weak level of support	2387 (6.8)	1.09 (0.96- 1.23)	1.09 (0.97- 1.24)	-0.36 (-0.57- -0.15)	-0.36 (-0.58- -0.15)
Low strain					

Strong level of support (ref)	342 (6.2)	1.00	1.00		
Weak level of support	845 (6.5)	1.06 (0.91- 1.21)	1.09 (0.96- 1.25)		
Passive					
Strong level of support	6781 (7.9)	1.27 (1.13- 1.43)	1.25 (1.11- 1.41)		
Weak level of support	3686 (7.5)	1.19 (1.06- 1.34)	1.21 (1.08- 1.37)	-0.13 (-0.29- 0.03)	-0.11 (-0.23 – 0.02)
**Secondary**					
Active					
Strong level of support	3480 (4.3)	1.01 (0.95- 1.08)	1.03 (0.97- 1.09)		
Weak level of support	4908 (4.7)	1.10 (1.04- 1.17)	1.11 (1.04- 1.17)	0.30 (-0.05- 0.11)	0.03 (-0.05 – 0.10)
High strain					
Strong level of support	2218 (7.2)	1.65 (1.54- 1.77)	1.58 (1.47- 1.68)		
Weak level of support	4048 (5.5)	1.27 (1.19- 1.34)	1.20 (1.12- 1.27)	-0.43 (-0.54- -0.31)	-0.36 (.0.45- -0.26)
Low strain					

Strong level of support (ref)	1653 (4.2)	1.00	1.00		
Weak level of support	2632 (4.6)	1.04 (0.98- 1.11)	1.05 (0.98- 1.12)		
Passive					
Strong level of support	14 817 (6.2)	1.40 (1.33- 1.48)	1.33 (1.27 1.40)		
Weak level of support	6942 (6.1)	1.38 (1.31- 1.47)	1.34 (1.26- 1.41)	-0.04 (-0.12- 0.04)	-0.03 (-0.09- 0.03)
**University**					
Active					
Strong level of support	2909 (2.9)	1.11 (1.04- 1.17)	1.07 (1.01- 1.14)		
Weak level of support	5549 (3.1)	1.18 (1.12- 1.24)	1.09 (1.04- 1.15)	0.09 (-0.02- 0.21)	0.08 (-0.02- 0.19)
High strain					
Strong level of support	891 (4.9)	1.79 (1.64- 1.94)	1.68 (1.54- 1.82)		
Weak level of support	2172 (4.0)	1.46 (1.37- 1.55)	1.28 (1.20- 1.36)	-0.32 (-0.49- -0.15)	-0.25 (-0.39- -0.12)
Low strain					

Strong level of support (ref)	2185 (2.7)	1.00	1.00		
Weak level of support	386 (2.5)	0.94 (0.94- 1.05)	0.93 (0.82- 1.04)		
Passive					
Strong level of support	2021 (4.4)	1.59 (1.49- 1.69)	1.47 (1.38- 1.57)		
Weak level of support	633 (4.3)	1.52 (1.38- 1.67)	1.38 (1.26- 1.53)	-0.01 (-0.19- 0.16)	-0.01 (-0.14- 0.11)

Relative risk due to interaction (RERI) = $HR_{11} – HR_{10} – HR_{01} + 1$.

Attributable proportion (AP) = RERI/HR_{11}

Crude: no adjustments

Adjusted: adjusted for age, country of birth, civil status, presence of children under 18 years in the household, , prior mental health problems, for childhood SEP, parents' highest level of education, and parents' mental and alcohol-related health problems

Supplementary Table S8. Crude and adjusted hazard ratios (HR) with 95% confidence intervals (CI) for the association of job strain and social support on the outcome of alcohol-related health problems for *women*, stratified by education

	Number of events n(%)	Crude (95% CI)	Adjusted (95% CI)	RERI (95% CI)	AP
**Primary**					
Active					
Strong level of support	240 (3.3)	0.91 (0.75- 1.10)	0.83 (0.69- 1.01)		
Weak level of support	336 (3.5)	1.00 (0.84- 1.19)	0.92 (0.78- 1.09)	0.21 (-0.14- 0.44)	0.23 (-0.02- 0.48)
High strain					
Strong level of support	424 (4.3)	1.23 (1.04- 1.45)	1.06 (0.90- 1.25)		
Weak level of support	245 (4.0)	1.14 (0.94- 1.38)	0.99 (0.82- 1.26)	0.05 (-0.20- 0.31)	0.05 (-0.20- 0.30)
Low strain					

Strong level of support (ref)	291 (3.4)	1.00	1.00		
Weak level of support	260 (3.8)	1.03 (0.85- 1.25)	0.87 (0.72- 1.06)		
Passive					
Strong level of support	2087 (3.3)	0.93 (0.81- 1.06)	0.89 (0.78- 1.02)		
Weak level of support	2042 (4.0)	1.09 (0.95- 1.25)	1.02 (0.89- 1.17)	0.26 (0.09- 0.43)	0.25 (0.07- 0.44)
**Secondary**					
Active					
Strong level of support	1241 (2.3)	0.99 (0.91- 1.08)	0.95 (0.87- 1.03)		
Weak level of support	1510 (2.3)	0.99 (0.91- 1.07)	0.96 (0.88- 1.04)	-0.30 (-0.41- -0.15)	-0.29 (-0.42- -0.16)
High strain					
Strong level of support	4678 (2.9)	1.20 (1.12- 1.28)	1.12 (1.05- 1.20)		
Weak level of support	880 (3.3)	1.41 (1.29- 1.56)	1.27 (1.16- 1.40)	-0.14 (0.28- 0.00)	-0.11 (-0.22 – 0.01)
Low strain					

Strong level of support (ref)	1238 (2.3)	1.00	1.00		
Weak level of support	1634 (3.5)	1.46 (1.34- 1.58)	1.29 (1.19- 1.40)		
Passive					
Strong level of support	5368 (2.6)	1.07 (1.01- 1.15)	1.03 (0.96- 1.10)		
Weak level of support	4403 (2.8)	1.50 (1.40- 1.61)	1.32 (1.23- 1.41)	-0.00 (-0.11- 0.10)	-0.00 (-0.09 – 0.08)
**University**					
Active					
Strong level of support	1164 (1.7)	1.02 (0.91- 1.15)	0.99 (0.88- 1.12)		
Weak level of support	5322 (1.7)	0.95 (0.86- 1.06)	0.92 (0.83- 1.02)	-0.02 (-0.17- 0.12)	-0.03 (-0.19 – 0.14)
High strain					
Strong level of support	710 (2.2)	1.28 (1.13- 1.46)	1.16 (1.02- 1.32)		
Weak level of support	1783 (2.0)	1.16 (1.03- 1.29)	1.09 (0.97- 1.22)	-0.02 (-0.19- 0.15)	-0.02 (-0.18 – 0.14)
Low strain					

Strong level of support (ref)	426 (1.7)	1.00	1.00		
Weak level of support	557 (1.8)	1.01 (0.88- 1.16)	0.95 (0.83- 1.09)		
Passive					
Strong level of support	836 (2.2)	1.23 (1.09- 1.40)	1.16 (1.03- 1.32)		
Weak level of support	619 (2.7)	1.52 (1.33- 1.73)	1.32 (1.15- 1.50)	0.20 (0.01- 0.39)	0.15 (0.01 – 0.29)

Relative risk due to interaction (RERI) = $HR_{11} – HR_{10} – HR_{01} + 1$.

Attributable proportion (AP) = RERI/HR_{11}

Crude: no adjustments

Adjusted: adjusted for age, country of birth, civil status, presence of children under 18 years in the household, prior mental health problems, for childhood SEP, parents' highest level of education, and parents' mental and alcohol-related health problems
